# Using machine learning to predict student mathematics performance in six East Asian countries: evidence from PISA 2022

**DOI:** 10.3389/fpsyg.2026.1840049

**Published:** 2026-06-05

**Authors:** Denis Djekourmane, Wei Zhang, Millicent Aziku, Dongfang Wu, Yang Zhang

**Affiliations:** 1 Faculty of education, Shaanxi Normal University, Xi'an, China; 2 School of Educational Sciences, Changji University, Changji, China

**Keywords:** East Asia, machine learning, mathematics performances, PISA 2022, self-determination theory, self-efficacy

## Abstract

**Introduction:**

In the contemporary era of artificial intelligence and rapidly evolving knowledge systems, mathematics performance has become a critical competency for younger generations. Although the mathematical achievement of students in East Asian countries has attracted increasing scholarly attention, studies employing machine learning techniques to examine the combined determinants of their success remain limited.

**Methods:**

This study evaluates six machine learning models-Random Forest, LightGBM, XGBoost, AdaBoost, Elastic Net, and Linear Regression to identify the most accurate algorithm for predicting mathematics performance among students from six high-performing East Asian countries/economies participating in the Programme for International Student Assessment (PISA) 2022. A sample of 26,969 fifteen-year-old students was analyzed. Following model selection, a post hoc feature selection procedure was applied, retaining the 24 most influential predictors from an initial set of 62 variables to ensure analytical parsimony while preserving model performance. SHapley Additive exPlanations (SHAP) values and SHAP interaction analyses were employed to quantify the magnitude, direction, and heterogeneity of each predictor's contribution at the individual level, including nonlinear relationships.

**Results:**

XGBoost emerged as the optimal model, demonstrating superior predictive accuracy (*R*^2^ = 0.5758, RMSE = 65.06) and explaining approximately 57.03% of the variance in mathematics achievement. Mathematics self-efficacy was identified as the most dominant predictor, exerting a substantially larger effect than all other variables, followed by participation in extracurricular activities before school and weekly mathematics instructional time. Affective, behavioral, and instructional factors consistently outperformed structural and socioeconomic variables in predictive importance.

**Discussion and conclusion:**

These findings underscore the central role of student-proximal determinants in mathematics achievement within Confucian Heritage Culture educational contexts. Interpreted through the lens of self-determination theory, the results carry important implications for educational policy and practice, particularly in prioritizing self-efficacy development, optimizing instructional time, and promoting equitable learning environments. The study also contributes theoretically and offers directions for future research.

## 1 Introduction

Mathematical ability has emerged as an indispensable competency in the contemporary knowledge economy, serving not merely as an academic discipline but also as a foundational literacy for navigating an increasingly data-driven, technologically mediated world (Acar, 2023; Adhikari et al., 2023; Aksu et al., 2017). During the Fourth Industrial Revolution, marked by artificial intelligence (AI), machine learning, automation, and big data analytics, mathematical proficiency serves as a crucial skill that determines access to high-value careers in science, technology, engineering, and mathematics (STEM) fields (Boman, 2022; Liu et al., 2026). Beyond vocational utility, mathematical reasoning cultivates critical thinking, problem-solving, and logical analysis, cognitive competencies essential for informed citizenship, financial literacy, and adaptive decision-making in complex societal contexts (Schoenfeld, 2016).

International comparative assessments, particularly the Programme for International Student Assessment (PISA) developed by the Organization for Economic Co-operation and Development (OECD), have consistently documented substantial cross-national disparities in mathematics achievement, raising concerns about educational equity and national competitiveness (OECD, 2024). Mathematics performance gaps carry long-term socioeconomic consequences. Scholars have mentioned that students with weak mathematical foundations tend to face diminished educational trajectories, constrained labor market prospects, and reduced lifetime earnings (Hanushek and Woessmann, 2015). At the national level, aggregate mathematical proficiency is associated with economic productivity, innovation capacity, and technological advancement (Glewwe and Muralidharan, 2016; Kim and Lee, 2010). Consequently, understanding the determinants of mathematics performance, and developing evidence-based interventions to enhance mathematics outcomes constitutes a priority for educational research and policy worldwide.

Over the past decades, students from East Asian education systems have consistently dominated international mathematics assessments. These students often achieve the highest mean scores and smallest achievement gaps in PISA, Trends in International Mathematics and Science Study (TIMSS), and other large-scale evaluations (OECD, 2023; Mullis et al., 2020). Since PISA started in 2000, countries and districts like Singapore, Hong Kong, Japan, Korea, Chinese Taipei, and Macao have consistently been at the top of the performance charts, often outperforming Western countries by 1–2 years of schooling (Jerrim, 2015, 2016). In PISA 2022, the six education systems included in the present study, namely Korea, Singapore, Japan, Chinese Taipei, Hong Kong, and Macao collectively accounted for 26,969 students, representing approximately 89% of the top-performing PISA participants globally (OECD, 2023). This sustained excellence has generated extensive scholarly inquiry into the “East Asian mathematics education paradox” (Jerrim, 2015; Leung, 2001).

Researchers have identified multiple contributing factors to mathematics performance in East Asian countries. Cultural and societal factors are among the most frequently discussed. Specifically, previous studies reported that Confucian heritage cultures emphasize diligence, perseverance, and respect for education, fostering high parental expectations, intensive academic effort, and societal valorization of scholarly achievement (Djekourmane et al., 2025; Tan, 2017). In this context, mathematics is culturally constructed as a domain where success derives from sustained and collective effort rather than innate and individual ability, promoting growth mindsets and resilience in the face of difficulty (Andersen and Nielsen, 2016; Kapasi and Pei, 2022; Yeager and Dweck, 2012). Moreover, adaptive instructional practices are frequently highlighted. East Asian mathematics pedagogy emphasizes deep conceptual understanding through coherent curriculum sequencing, systematic problem variation, and extensive practice consolidating core procedures (Jhang and Lee, 2018; Ma and Ma, 1999; Stokes, 2009). In that context, classroom instruction frequently employs the “structured problem-solving” model, wherein teachers guide students through carefully sequenced examples that build progressively toward mastery of complex concepts (Fung et al., 2017). Additionally, shadow education systems have also been reported, with supplementary private tutoring being pervasive and participation rates exceeding 70% in some contexts (Bray and Lykins, 2012). This parallel educational infrastructure extends instructional time, provides targeted remediation, and intensifies competitive preparation for high-stakes examinations. Systemic coherence is also considered a dominant factor, referring to the alignment and integration of curriculum, assessment, teacher preparation, and policy, which creates institutional conditions conducive to instructional effectiveness (OECD, 2018).

However, this aggregate excellence masks considerable within-system heterogeneity. Even in top-performing East Asian countries and economies, substantial proportions of students fail to achieve proficiency benchmarks, and achievement gaps linked to socioeconomic status, gender, and immigrant background persist (OECD, 2018, 2023). Moreover, high-performing systems confront ongoing challenges, including excessive academic pressure, mental health concerns, and curricular narrowing (Ahmed et al., 2020; Deb et al., 2016). Understanding the multiple and interlinked individual-level and contextual factors that determine achievement within these elite systems remains an urgent research priority. This is because policy decisions, practical interventions, and future research require evidence-based insights grounded in updated international data. Although previous studies have provided some insights in this context, they remain fragmented rather than holistic (Boman, 2022; Jhang and Lee, 2018).

To better understand these complex relationships, this study is guided by self-determination theory (SDT), developed by (Deci and Ryan 1985). SDT posits that learning and performance are shaped by the fulfillment of three fundamental psychological needs: autonomy (a sense of volition and agency), competence (a sense of effectiveness and capability), and relatedness (a sense of connection to others). When these needs are supported, individuals are more likely to develop intrinsic motivation, sustain engagement, and achieve higher levels of performance (Ryan and Deci, 2020). Recent studies further confirm that need satisfaction significantly predicts student engagement, academic achievement, and learning outcomes across both traditional and digital environments (Alturki and Aldraiweesh, 2024; Fàbregues et al., 2025; Jones et al., 2025). In the context of mathematics achievement, students' outcomes are not solely determined by cognitive ability, but also by the extent to which their learning environments support these psychological needs, as well as their resulting motivation, persistence, and engagement (Abdulla Alabbasi et al., 2023; Acosta-Gonzaga, 2023). For example, cultural expectations, teaching practices, and access to shadow education can be understood as contextual factors that either support or constrain need satisfaction, while students' confidence and effort reflect competence and motivated engagement (Alamri, 2023; Hau and Ho, 2010). By integrating SDT with machine learning approaches, this study aims to capture the complex, non-linear interactions among these factors and identify key predictors of mathematics performance in six high-performing East Asian education systems using PISA 2022 data.

Previous studies have tended to apply machine learning (ML) to explore academic performance among East Asian and global student populations (Jerrim, 2015; Liu et al., 2026). To the best of our knowledge, studies using PISA 2022 data to examine student mathematics performance across six top-performing countries remain scarce. Moreover, the novelty of the present study extends beyond its regional focus. It lies in the integration of a comprehensive set of predictors, a robust methodological pipeline, and the application of SHAP and SHAP interaction analyses for model interpretability, all grounded in the theoretical framework of SDT. By combining advanced analytical techniques with a strong theoretical foundation, this study aims to provide a more comprehensive understanding of student achievement and to offer meaningful implications for educational research, policy, and practice.

## 2 Literature review

### 2.1 Theoretical framework

Informed by a range of theoretical perspectives, including social cognitive theory, cultural capital theory and socioecological theory, this study is mainly grounded in self-determination theory (SDT) (Deci and Ryan, 1985; Ryan and Deci, 2020). Others theories such as social cognitive theory (Bandura, 1997), expectancy-value theory (Eccles and Wigfield, 2023), cultural capital theory (Bourdieu, 1979), and so forth are used as complementary theoretical lenses. Developed by Deci and Ryan, SDT provides a comprehensive framework for understanding human motivation, learning, and performance and has been widely applied in educational research (Reeve, 2012; Shogren et al., 2025). SDT posits that human behavior and optimal functioning are driven by the satisfaction of three basic psychological needs: autonomy (the need to experience volition and self-direction), competence (the need to feel effective and capable), and relatedness (the need to feel connected to others) (Ahmadi et al., 2023; Deci and Ryan, 1985). These needs are universal and essential for fostering intrinsic motivation, sustained engagement, and high-quality learning. When learning environments support these needs, individuals are more likely to internalize motivation and achieve better outcomes.

In this context, SDT offers a robust lens for examining student achievement. Personal factors can be understood in terms of competence beliefs (e.g., self-efficacy), intrinsic motivation, and attitudes toward mathematics. Among these, competence-related beliefs have been consistently identified as strong predictors of academic performance, influencing students' persistence, effort, and resilience when facing challenging tasks (Arens et al., 2022; Beatson et al., 2020; Christy and Mythili, 2020). Behavioral factors reflect students' motivated engagement, including time spent on tasks, active participation in classroom activities, and involvement in supplementary learning practices such as private tutoring (Djekourmane et al., 2025). Environmental factors encompass instructional quality, teacher support, parental expectations, and broader cultural norms, all of which contribute to the satisfaction or frustration of students' psychological needs (Collie et al., 2024; Scherer et al., 2023). Existing literature highlights that mathematics achievement is shaped by the interaction of these motivational and contextual dimensions rather than isolated variables. Research based on international large-scale assessments such as PISA demonstrates that students' motivation, learning strategies, and socio-economic backgrounds jointly contribute to performance outcomes (Hernández-Torrano and Courtney, 2021; OECD, 2024). Furthermore, studies on East Asian education systems emphasize the role of cultural values, structured pedagogy, and extended learning opportunities in supporting competence development and shaping motivational orientations (Jerrim, 2015; Leung, 2001). These findings align with SDT's assumption that learning outcomes emerge from the dynamic interplay between psychological need satisfaction and contextual influences.

In addition, recent research integrating educational data mining and learning analytics has demonstrated the importance of examining complex and non-linear relationships among multiple predictors of student performance (Cerezo et al., 2024). Machine learning approaches, such as random forest and clustering techniques, have been increasingly applied to identify key determinants of academic success and uncover hidden patterns in large-scale educational datasets (Algarni, 2016; Aulakh et al., 2023). These approaches complement SDT by enabling the modeling of interactions between psychological needs, motivation, and contextual factors in a data-driven manner. By adopting SDT as the theoretical framework, this study would provide a structured approach to understanding the motivational mechanisms underlying mathematics performance. Furthermore, integrating SDT with machine learning techniques allows for the identification of key predictors and non-linear interactions among variables, offering a more comprehensive and data-driven explanation of student achievement in high-performing East Asian education systems.

### 2.2 Determinants of students mathematics achievement

Several decades of educational research have established that mathematics achievement is multiply determined by the complex interplay of individual, familial, instructional, and systemic factors (Byrnes and Miller, 2007; Hattie, 2010). Comprehensive models situated student performance within nested ecological contexts, individual cognitive and affective attributes embedded within classroom instruction, school climate, family resources, and broader societal structures (Bronfenbrenner, 2009; Creemers and Kyriakides, 2010). The following subsections synthesize empirical evidence on major predictor domains relevant to the present study.

#### 2.1.1 Student affective and motivational factors

As mentioned above, Bandura's (1997) social cognitive theory posits self-efficacy, individuals' beliefs in their capabilities to execute actions required to achieve specific goals, as a central determinant of motivation, effort allocation, and achievement. In mathematics education, self-efficacy consistently emerges as one of the strongest predictors of performance across diverse cultural contexts (Aksu et al., 2017; Alamri, 2023; OECD, 2016b; Pajares, 1992). Empirical past evidence indicated moderate-to-strong correlations between mathematics self-efficacy and achievement, with bidirectional causality: prior success enhances self-efficacy, which in turn promotes persistence, cognitive engagement, and subsequent performance gains (Marsh et al., 2019; Marsh and Martin, 2011; Rønnow-Rasmussen and Zimmerman, 2005). PISA data reveal robust self-efficacy-achievement associations across all participating countries, but they are particularly pronounced in East Asian systems where growth mindsets predominate (OECD, 2023). Students with high self-efficacy employ more sophisticated problem-solving strategies, exhibit greater willingness to tackle challenging problems, and recover more effectively from setbacks (Schunk and Pajares, 2009). Conversely, low self-efficacy triggers mathematics anxiety, avoidance behaviors, and learned helplessness, creating vicious cycles of underachievement (Ashcraft and Krause, 2007).

Furthermore, SDT (Ryan and Deci, 2020) distinguishes intrinsic motivation (engagement driven by inherent interest) from extrinsic motivation (engagement driven by external rewards or pressures). Students intrinsically motivated toward mathematics demonstrate deeper cognitive engagement, greater conceptual understanding, and sustained achievement gains (Corpus et al., 2009; Gottfried et al., 1998). However, previous studies indicated that intrinsic motivation toward mathematics is paradoxically lower in East Asian systems compared to Western countries. This suggests that achievement is sustained through extrinsic pressures (examinations, parental expectations) rather than inherent interest, a phenomenon termed the “motivation–achievement paradox” (OECD, 2013).

In addition, mathematics anxiety, characterized by the feelings of tension, apprehension, and fear when confronting mathematical tasks, affects students globally and correlates negatively with achievement (Dowker et al., 2016; Foley et al., 2022). Neuroscientific research indicated that mathematics anxiety hijacks working memory resources through intrusive worry, impairing cognitive processing during problem-solving (Ramirez et al., 2013). Longitudinal past study also documented anxiety's debilitating effects on course enrollment, persistence in STEM pathways, and career aspirations (Ma and Xu, 2004).

#### 2.1.2 Time investment and engagement

The “opportunity to learn” hypothesis posits that achievement is fundamentally constrained by the quantity and quality of instructional exposure (Cairns and Areepattamannil, 2022; Genc and Colakoglu, 2021; Schmidt and McKnight, 2012). Cross-national comparison studies revealed weak-to-moderate correlations between allocated instructional time and achievement, suggesting that time's impact is mediated by instructional quality, curriculum coherence, and student engagement (Lavy, 2015; Rivkin and Schiman, 2015). Some studies documented diminishing marginal returns or even negative effects at very high instructional intensities, potentially reflecting curricular redundancy, student fatigue, or displacement of other valuable learning activities (Patall et al., 2008).

Past empirical evidence also indicated positive but heterogeneous homework-achievement associations, with effect sizes varying by grade level (stronger in secondary than elementary), subject area, and homework design (Cooper et al., 2006). In other words, high-quality homework that reinforces classroom learning, provides cognitive challenges, and offers timely feedback enhances achievement, whereas excessive, rote, or poorly designed homework may induce stress, undermine intrinsic motivation, and consume time better allocated to rest or extracurricular enrichment (Dettmers et al., 2010; Trautwein et al., 2009). Researchers showed that East Asian students reported substantially higher homework burdens than Western counterparts, raising questions about optimal dosage and diminishing returns (Mullis et al., 2020). Moreover, involvement in academically focused extracurricular activities (such as academic clubs, competitions, and enrichment programs) was positively correlated with achievement, possibly indicating unquantified student motivation, parental support, or cognitive stimulation (Dumais, 2006; Richards et al., 2026). However, excessive extracurricular involvement may also crowd out study time, sleep, and leisure, undermining wellbeing and academic performance (Fredricks and Eccles, 2006).

#### 2.1.3 Instructional quality and pedagogical practices

Student cognitive activation encompasses instructional methodologies that compel students to engage in profound thinking, establish connections among concepts, rationalize their reasoning, and apply knowledge to unfamiliar problems (Blömeke et al., 2020; Lipowsky et al., 2009; Shogren et al., 2025). PISA's cognitive activation index, derived from student reports of classroom practices such as explaining reasoning, solving complex problems, and relating content to real-world contexts, predicts achievement across countries (OECD, 2016a). Past studies confirmed that classrooms emphasizing conceptual understanding, mathematical reasoning, and problem-solving produce superior learning outcomes compared to procedural, rote-focused instruction (Boaler, 2002; Hiebert and Grouws, 2006). Moreover, effective mathematics teaching is considered to integrate clear explanations, high-quality questioning, formative assessment, differentiated support, and opportunities for student reasoning (Charalambous and Praetorius, 2018). Teacher pedagogical content knowledge (PCK), specialized knowledge of how to represent mathematical concepts, anticipate student difficulties, and design effective learning sequences, is also a critical determinant of instructional quality and student achievement (Baumert et al., 2010; Hill et al., 2005). However, researchers raised concern that measuring instructional quality via student self-reports (as in PISA) introduces measurement error and social desirability bias, attenuating observed associations (Wagner et al., 2016).

Furthermore, contemporary mathematics education increasingly emphasizes “21st-century competencies” that include critical thinking, problem-solving, collaboration, digital literacy, and creativity, aligned with knowledge economy demands (Imjai et al., 2024; Voogt and Roblin, 2012). PISA 2022 introduced measures of exposure to these competencies, hypothesizing positive associations with achievement. However, empirical evidence remains mixed: some studies document synergies between 21st-century pedagogies and achievement (Bayley, 2022), while others warn of potential trade-offs if such approaches displace foundational skill development (Kirschner et al., 2006), suggesting that a balanced approach that integrates both 21st-century competencies and foundational skills may be necessary for optimal educational outcomes.

#### 2.1.4 Socioeconomic and family background

Socioeconomic status (SES), typically operationalized via parental education, occupation, and household resources is among the most consistent predictors of educational achievement worldwide (Djekourmane et al., 2025; Sirin, 2005). The PISA index of economic, social, and cultural status (ESCS) exhibits moderate-to-strong correlations with mathematics achievement across countries, with SES gradients steeper in unequal societies (OECD, 2016b). Socioeconomic disparities operate through multiple pathways: differential access to educational resources (books, tutoring, technology), parental involvement and educational expectations, linguistic capital, neighborhood quality, and exposure to academic stress and trauma (Bradley and Corwyn, 2002; Duncan and Murnane, 2011). In East Asia, the SES gradients in education systems are relatively flat compared to those in other countries. This finding could mean that education is fairer or that there are stronger compensatory mechanisms (An Le et al., 2021; OECD, 2018). However, within-system SES effects remain substantial, and recent evidence indicates rising inequality associated with shadow education access, residential segregation, and neoliberal education reforms (Byun et al., 2012; Perry et al., 2022).

In connection with these findings, parental education level, particularly maternal education, predicts student achievement net of income and occupation, reflecting parents' capacity to support learning, model academic values, and navigate educational systems (Andrade and Fernandes, 2023; Demaray and Jenkins, 2011). Specifically, students whose parents expect postsecondary attainment achieve higher outcomes even when controlling for prior performance and SES (Benner and Mistry, 2007; Yamamoto and Holloway, 2010). In East Asian contexts, parental expectations are universally high, potentially compressing variance and attenuating measured effects (Hou et al., 2023; Jhang and Lee, 2018). Furthermore, home possessions and cultural capital pertaining to educational resources, such as books, computers, desks, and tranquil study environments, enhance learning and exhibit a positive correlation with academic achievement (Djekourmane et al., 2025; Morkevicius et al., 2022). Bourdieu's (1979) cultural capital theory also posits that middle-class families transmit cultural knowledge, linguistic codes, and academic dispositions valued in schools, conferring advantages independent of material resources. PISA measures cultural possessions (e.g., art, classic literature) and finds positive associations with achievement, though effects vary by cultural context (Andersen and Jaeger, 2015; Balzer et al., 2025).

Access to digital devices such as computers, tablets, and the internet is increasingly posited as essential for 21st-century learning, facilitating access to online resources, educational software, and digital literacy development (Warschauer and Matuchniak, 2010). However, past studies revealed complex, non-linear associations: moderate technology use correlates positively with achievement, but excessive use (particularly for entertainment) correlates negatively, reflecting displacement of study time or shallow information processing (Bano et al., 2018). The present study includes digital device access among predictors, challenges techno-determinist assumptions, and is valuable.

#### 2.1.5 School climate and behavioral factors

Grade repetition, requiring students to repeat a grade due to insufficient performance, is controversial, with proponents arguing it provides additional time for skill consolidation and critics highlighting stigmatization, disengagement, and opportunity costs (Bulut, 2021). Existing evidence indicated predominantly negative long-term effects: retained students exhibit lower achievement, higher dropout rates, and worse socio-emotional outcomes compared to promoted peers (Allen et al., 2009; Jimerson et al., 2002). Past studies also document strong negative correlations between retention and achievement, though causal directionality remains ambiguous; low achievement triggers retention, but retention may also further depress achievement.

Similarly, chronic absenteeism, characterized by missing school days, predicts lower achievement, diminished engagement, and elevated dropout risk (Balfanz and Byrnes, 2012; Gottfried, 2014). Even sporadic absences accumulate to produce measurable achievement deficits, reflecting lost instructional time, disrupted learning continuity, and peer effects (Aucejo and Romano, 2016). East Asian systems report low truancy rates, likely reflecting strong school discipline, parental monitoring, and cultural norms valuing attendance (OECD, 2019). In addition, school safety perceptions and bullying victimization negatively predict achievement, operating through compromised psychological wellbeing, fear-induced absenteeism, and cognitive distraction (Nakamoto and Schwartz, 2010; Strøm et al., 2013). Researchers indicated that students reporting frequent bullying scored significantly lower in mathematics, with effects particularly pronounced for relational aggression (OECD, 2017). The present study's inclusion of school safety variables addresses this underexplored dimension in East Asian contexts.

Previous studies also revealed that positive teacher–student relationships, characterized by warmth, support, fairness, and responsiveness, foster student motivation, engagement, and achievement (Cornelius-White, 2007; Roorda et al., 2011). The literature often documents small-to-moderate positive associations between relationship quality and academic achievement, with effects mediated by student self-efficacy, classroom participation, and help-seeking (Wentzel et al., 2010). Contextually, East Asian classrooms traditionally emphasize hierarchical teacher authority, potentially constraining relationship intimacy, though recent reforms promote more student-centered pedagogies (Watkins, 2001), which may enhance the quality of teacher–student relationships and subsequently improve student motivation and achievement.

#### 2.1.6 Structural and demographic factors

Grade progression represents developmental maturation, cumulative instructional exposure, and curricular advancement. The present study includes grade level among predictors to confirm its importance to student academic achievement. Moreover, international large-scale assessments consistently document gender gaps favoring males in mathematics, though magnitudes vary by country and have narrowed over time (Else-Quest et al., 2010; Lindberg et al., 2010). East Asian systems exhibit relatively small or negligible gender gaps compared to others, potentially reflecting egalitarian cultural norms or universal expectations for mathematical competence in these countries (OECD, 2023). Stereotype threat, self-efficacy differences, and gendered socialization contribute to observed gaps (Gunderson et al., 2012). Further, immigrant students, particularly first-generation immigrants, often underperform native peers due to linguistic barriers, cultural dislocation, socioeconomic disadvantage, and discrimination (OECD, 2018). However, East Asian immigrant communities often exhibit achievement advantages, reflecting selective migration, strong family educational values, and ethnic capital mobilization (Zhou and Kim, 2006). The present study's East Asian sample likely captures this phenomenon to expand our understandings.

Despite extensive research, several methodological and contextual limitations constrain understanding of mathematics achievement determinants. Traditional regression-based approaches assume linear, additive relationships between predictors and outcomes, potentially misspecifying complex non-linear, interactive, and threshold effects (e.g., diminishing returns to study time and conditional effects of self-efficacy moderated by instructional quality). Researchers typically select predictor subsets based on theoretical priors or statistical parsimony criteria, risking omitted variable bias, multicollinearity, and unstable coefficient estimates when predictors are correlated (Vanhove, 2021). Comprehensive past analyses indicated that traditional regression models typically explain 40%−60% of variance in mathematics achievement (Arnold, 2011), leaving substantial unexplained variance. Weak predictive power undermines utility for early identification, targeted intervention, and personalized learning systems. Traditional methods struggle to uncover higher-order interactions, non-monotonic effects, and latent patterns (e.g., distinct student profiles exhibiting equifinality, multiple pathways to success). Some studies may be constrained by the limited existing variables in the analyzed dataset; others may overlook the multiple and complex factors driving mathematics performance.

### 2.2 Machine learning

Machine learning (ML) is a branch of artificial intelligence (AI) in which computer algorithms learn patterns from data and improve their predictions over time. This approach offers important advantages over traditional statistical methods, particularly when dealing with complex educational data (Jordan and Mitchell, 2015; Liu et al., 2026). Unlike conventional research methods that test pre-specified theories, ML focuses on discovering patterns, making accurate predictions, and producing results that hold up on new, unseen data, making it well suited for studying the many interacting factors that shape student learning (Aguilar Sanchez et al., 2025; Jordan and Mitchell, 2015).

Among the most powerful ML approaches are ensemble methods, techniques that combine many individual models to produce stronger predictions than any single model could achieve. Examples include gradient boosting algorithms such as XGBoost and LightGBM, as well as random forests. These methods have consistently outperformed traditional regression models in predicting educational outcomes, often explaining substantially more variation in student performance (Alsariera et al., 2022; Tahri et al., 2026). A key advantage of these approaches is their ability to automatically detect non-linear relationships and complex interactions between variables, without researchers needing to specify these patterns in advance. They also include built-in tools for selecting the most relevant predictors from large datasets, helping to avoid common problems such as redundant variables and overfitting, where a model performs well on training data but poorly on new data.

Tree-based ensemble models are also well suited to the kinds of data challenges commonly found in education research, including unusual values, skewed distributions, and missing responses (Bernardo et al., 2023). Supporting techniques such as Isolation Forest, which identifies and removes unusual data points, and RobustScaler, which standardizes variables in a way that is less sensitive to extreme values, further improve model reliability. Beyond building strong models, ML also uses rigorous testing procedures, such as splitting data into training and testing sets and using cross-validation, to confirm that findings generalize beyond the original sample, a quality check that is often missing in traditional educational research (Hastie et al., 2009).

A particularly important recent development is the use of SHAP (SHapley Additive exPlanations) values to make ML models more transparent and interpretable (Shapley, 2016). Rather than treating the model as a “black box,” SHAP explains how much each variable contributes to a prediction, in which direction, and whether its effect changes across different students, providing clear, actionable insights for researchers and policymakers alike (Lundberg and Lee, 2017). Recent studies have demonstrated ML's practical value in predicting mathematics achievement and identifying students at risk of underperformance, enabling schools to intervene earlier and more effectively (Kizilcec et al., 2020; Liu et al., 2026). Analyses of PISA data using ML have further revealed cross-national patterns in what predicts student success, patterns those traditional methods had previously missed offering more targeted guidance for education policy (Hussain et al., 2018).

Despite these advances, relatively few studies have applied ML to large-scale international datasets to examine what drives mathematics performance in top-performing East Asian education systems. This study addresses that gap by applying ML methods to PISA 2022 data to identify the key factors shaping the mathematics achievement of 15-year-old students across these high-performing contexts.

### 2.3 The present study

Despite extensive research on mathematics excellence in East Asia, critical knowledge gaps remain. Most previous studies concentrate on overall system-level performance, thereby concealing significant intra-system variation. In addition, existing research typically examines narrow subsets of predictors, such as socioeconomic status or affective factors in isolation, while neglecting the inherently multifactorial nature of academic achievement (Jerrim, 2015). Moreover, despite the widespread documentation of correlational relationships, the identification of high-leverage factors that can guide targeted interventions has received limited attention. Advanced approaches, such as feature importance rankings and SHAP analysis, would offer a more precise means of addressing this limitation.

To bridge these gaps, the present study applies a comprehensive ML pipeline to predict mathematics achievement among 26,718 students across six top-performing East Asian education systems (Korea, Singapore, Japan, Chinese Taipei, Hong Kong, and Macao) using PISA 2022 data. First, the study develops and validates high-accuracy ML models (XGBoost, LightGBM, and Random Forest), benchmarking their predictive performance against traditional linear regression. It further examines the relative importance of predictors spanning demographic, socioeconomic, affective, behavioral, instructional, and school-level domains. Second, SHAP analysis is employed to uncover non-linear relationships, directional effects, and interaction patterns, thereby enhancing the model's interpretability. Third, the study seeks to produce actionable evidence to guide educational policy and practice by pinpointing high-impact intervention targets.

This study would make several contributions. Methodologically, it illustrates the benefits of ML compared to conventional regression techniques by offering a replicable analytical framework that incorporates outlier detection, robust scaling, feature selection, hyperparameter optimization, and SHAP-based interpretability. Empirically, it offers a comprehensive, multidimensional analysis of mathematics achievement determinants within high-performing systems, shifting attention from cross-national comparisons to within-system heterogeneity. Practically, it translates predictive insights into policy-relevant recommendations and provides a transparent, extensible framework for future research. The study addresses the following research questions:

**RQ1:** What level of predictive accuracy can ML models achieve, and how do they compare with traditional regression approaches?

**RQ2:** Which predictors most strongly influence mathematics performance from six top performing countries/economies, and how do these align with existing theory and evidence?

**RQ3:** What non-linear and interaction effects characterize key determinants of achievement?

**RQ4:** What implications emerge for educational policy, instructional practice, and intervention design for regional and global?

## 3 Methods

### 3.1 Dataset and sample

**Figure 1:**
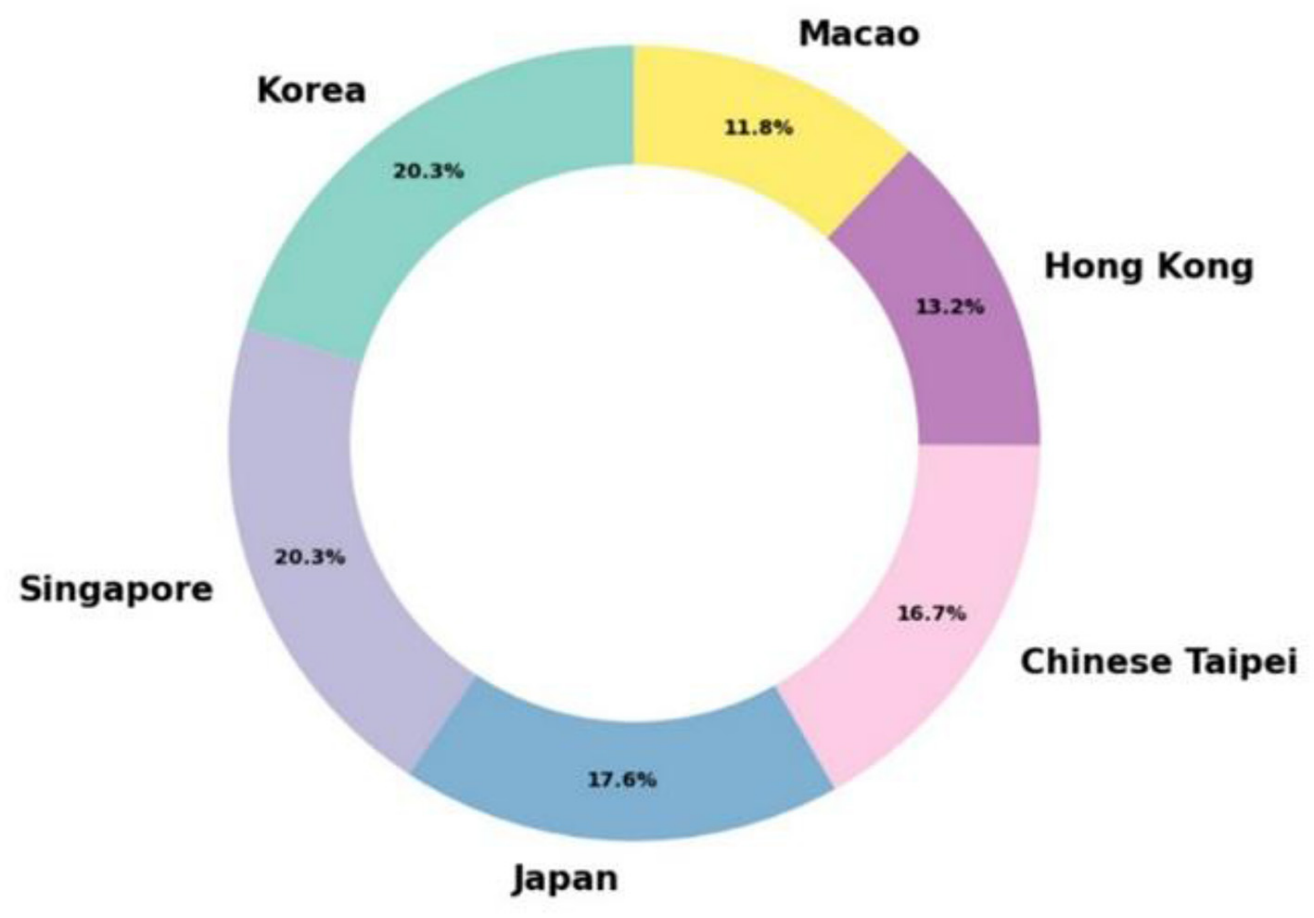
Countries distribution.

This study utilized data from the Programme for International Student Assessment (PISA) 2022, with a specific focus on mathematics achievement among 15-year-old students. The initial sample comprised 26,969 students drawn from six high-performing East Asian education systems: Korea (20.33%), Singapore (20.31%), Japan (17.61%), Chinese Taipei (16.67%), Hong Kong (13.24%), and Macao (11.84%). Following data cleaning procedures, including the removal of outliers, the final analytical sample consisted of 26,718 students (Figure 1 and Table 1).

**Table 1:** Countries distribution.

Country	Sample size	Percentage
Korea	5,483	20.33%
Singapore	5,477	20.31%
Japan	4,749	17.61%
Chinese Taipei	4,495	16.67%
Hong Kong	3,572	13.24%
Macao	3,193	11.84%
Total	26,969	100.00%

The dataset included 64 variables representing multiple dimensions of student and school characteristics. These variables encompassed student demographics, socioeconomic status (ESCS), parental education and qualifications, instructional quality, access to digital devices, school experiences, exposure to 21st-century skills, cognitive activation, and other indicators of mathematics performance.

### 3.2 Target variable

The dependent variable, *Mathematics Performance*, was operationalized as the mean of ten plausible values (PV1MATH-PV10MATH) provided in the PISA dataset. This approach accounts for the measurement uncertainty inherent in large-scale assessments and yields a more stable and reliable estimate of individual mathematics achievement than any single plausible value.

### 3.3 Data preprocessing and analytical process

Prior to model development, a structured preprocessing and analytical pipeline was implemented to ensure data quality, reproducibility, and methodological rigor. Initial data preparation, including variable recoding and harmonization, was conducted in *Stata (version 18.0)*. The dependent variable, mathematics performance, was derived from multiple plausible values provided in the PISA 2022 dataset. Consistent with established practices in large-scale educational assessments, these plausible values were aggregated into a single composite score to obtain a stable estimate of student ability (OECD, 2024). All subsequent analyses were performed in *Python 3.12* (Anaconda distribution, version 2024.10), using *scikit-learn (v1.4)* for preprocessing and evaluation, XGBoost for predictive modeling, and *SHAP (v0.45)* for model interpretability. This integrated pipeline enables reproducibility and facilitates the implementation of advanced ML techniques (Lones, 2024).

#### 3.3.1 Data training and split-test

To ensure unbiased model evaluation, the dataset was partitioned into training (80%) and testing (20%) subsets using a random split (random_state = 42). The training set (*n* = 21,575) was used for model development and hyperparameter tuning, while the test set (*n* = 5,394) was reserved exclusively for final performance evaluation. This separation is essential to prevent information leakage and to obtain an unbiased estimate of out-of-sample predictive performance (Sohil et al., 2022).

#### 3.3.2 Outlier detection and removal

Outlier detection was conducted prior to model training to enhance robustness and reduce the influence of anomalous observations. A two-stage approach was adopted:

- Univariate outliers: observations exceeding ±3.5 standard deviations from the mean of the outcome variable were removed, resulting in the exclusion of 1 observation (0.004%) (Hoaglin, 2013).
- Multivariate outliers: the isolation forest algorithm was applied to detect complex anomalies in high-dimensional space (Liu et al., 2008). The model was trained on the 20 highest-variance predictors using a contamination rate of 5% and max_samples = 256. This step identified and removed 250 observations (0.93%).

In total, 251 observations (~0.9%) were excluded, yielding a final analytical sample of 26,718 students. We deliberately applied two-stage isolation forest because of its suitability for high-dimensional, non-normally distributed educational datasets, with anomalous response patterns like in PIS data, where conventional distance-based outlier methods lose effectiveness due to the curse of dimensionality (Liu et al., 2008). Indeed, the total exclusion of 251 observations (~0.9%) from a sample of 26,969 students though could create bias from population-level, the percentage remains minimal.

#### 3.3.2 Missing data imputation and feature engineering

Data imputation: missing values were imputed using median imputation, which is robust to skewed distributions and less sensitive to extreme values and mostly recommended by researchers in ML (Little, 2024).

Feature scaling: all predictor variables were standardized using the RobustScaler, which scales features based on the interquartile range (IQR). This approach is preferable in the presence of outliers, as it reduces their influence compared to standard normalization techniques (Sohil et al., 2022).

Feature selection: to reduce dimensionality and improve model efficiency, SelectKBest with *f_regression* scoring was used to select the top 50 predictors. Feature selection helps mitigate multicollinearity and enhances generalization performance.

### 3.4 Machine learning models

A diverse set of machine learning models was evaluated to capture both linear and non-linear relationships within the data. Ensemble methods were prioritized due to their strong predictive performance in structured datasets (Tahri et al., 2026). To enhance accessibility, each model is described concisely with emphasis on its relevance to the research objectives rather than technical complexity. The following models were included:

1. XGBoost (Extreme Gradient Boosting): an efficient gradient boosting framework designed for high predictive performance and scalability.
2. LightGBM (Light Gradient Boosting Machine): a gradient boosting approach that utilizes histogram-based algorithms to improve computational efficiency.
3. Random forest: an ensemble method based on bagging, which constructs multiple decision trees to improve robustness and reduce overfitting.
4. AdaBoost: a boosting algorithm that iteratively focuses on misclassified or difficult observations to enhance model accuracy.
5. Elastic Net: a regularized linear model that combines L1 and L2 penalties, making it effective in handling multicollinearity.
6. Linear regression: a baseline model included to provide a benchmark for comparing the performance of more complex approaches.

All models were implemented using *scikit-learn* and their respective libraries, with a fixed random state (random_state = 42) to ensure reproducibility. This combination of models allows for a balanced comparison between interpretable linear approaches and more flexible non-linear methods.

### 3.5 Hyperparameter optimization

Hyperparameter tuning was conducted using 5-fold cross-validation on the training dataset, ensuring robust estimation of model performance and reducing the risk of overfitting (Sohil et al., 2022). In this procedure, the training data were partitioned into five equal subsets. For each iteration, fourfolds were used for model training and onefold for validation, such that each observation served as a validation instance exactly once. This approach improves the stability and reliability of performance estimates compared to a single validation split.

Hyperparameter search was implemented using a structured search strategy (e.g., *GridSearchCV*), with *R*^2^ selected as the primary scoring metric due to its interpretability as a measure of explained variance in continuous outcomes. All preprocessing steps (imputation, scaling, and feature selection) were embedded within the cross-validation pipeline and applied independently within each fold to prevent data leakage (Sohil et al., 2022).

### 3.6 Model evaluation

Following hyperparameter optimization, the final models were trained on the full training dataset and evaluated on the held-out test set. This approach ensures an unbiased assessment of model generalization. Performance was assessed using five complementary metrics:

1. *R*^2^ score: proportion of variance explained.
2. Root mean squared error (RMSE): average magnitude of prediction errors.
3. Mean absolute error (MAE): average absolute prediction errors.
4. Explained variance: proportion of variance explained without bias correction.
5. AUC-regression: area under the cumulative residual curve, calculated as:

$$\begin{array}{l} AUC\;=\;\overset{1}{\underset{0}{\int }}F\left(\frac{r\;}{r_{\text{max}}}\right)=dr \end{array}$$

where *r* denotes the absolute residuals observed in the sample, *F* represents the empirical cumulative distribution function (ECDF) of the normalized residuals.

### 3.7 SHAP analysis

Native feature importance scores from the best-performing model (based on mean decrease in impurity) were extracted and ranked. Further, SHapley Additive exPlanations (SHAP) values were computed on a random sample of 26,969 observations to quantify each feature's contribution to individual predictions. SHAP provides both global feature importance and directional impact (positive vs. negative influence on predictions).

## 4 Results

### 4.1 Descriptive statistics

Table 2 summarizes the means and standard deviations of the study variables, providing an overview of the sample characteristics and variability across key constructs. Students were predominantly in Grade 9 (M = 9.82, SD = 0.43), with a balanced gender distribution (M = 1.50, SD = 0.50). Further, substantial variability is observed in family background indicators. Parental education and qualifications exhibit notably large standard deviations (e.g., Mother_educ: *M* = 3.07, SD = 11.16; Father_educ: *M* = 3.93, SD = 14.33), suggesting considerable heterogeneity in parental educational attainment and possible coding dispersion. Language background (*M* = 1.21, SD = 2.69) and immigration status (*M* = 2.66, SD = 2.78) also indicate diversity within the sample.

**Table 2:** Descriptive statistics of key variables.

Variable	Mean	SD
Grade	9.82	0.43
Gender	1.50	0.50
Mother_educ	3.07	11.16
Father_educ	3.93	14.33
Language_bac	1.21	2.69
Math_Hour_week	7.49	13.56
Math_instruction_quality	24.95	36.64
Digital_device_for_school_work	42.37	46.67
Learning_school_closure	42.16	46.83
Immigration_status	2.66	2.78
Cognitive_activation_score	0.36	6.62
Exposure_21st_century_skills	5.33	7.60
Mathematics_performance	0.51	6.84
ESCS_homeposs	1.60	0.66
ESCS_Digital_deviceshome	1.55	1.72
Grade_repetition	1.88	36.80
Truancy	18.65	36.77
Absenteism	9.23	16.36
Student_Teacher_Relations_Qual	38.22	2.73
School_Sense_of_belonging	18.59	3.66
School_bulling	13.07	3.01
School_Safety	18.47	36.36
School_safety_risks	30.50	43.37
Extra_Activities_before_school	2.20	4.10
Extra_Activities_after_school	4.42	7.77
Online_activities_Time	4.30	7.15
Digital_device_usage_behaviors	19.05	3.80

Regarding learning-related variables, time spent on mathematics per week shows high variability (*M* = 7.49, SD = 13.56), indicating uneven levels of engagement. Similarly, instructional quality (*M* = 24.95, SD = 36.64) and access to digital devices for schoolwork (*M* = 42.37, SD = 46.67) display substantial dispersion, reflecting differences in educational environments. Exposure to 21st-century skills (*M* = 5.33, SD = 7.60) and cognitive activation (*M* = 0.36, SD = 6.62) further highlight variability in instructional practices. Mathematics performance (*M* = 0.51, SD = 6.84) demonstrates notable spread, suggesting considerable differences in student achievement. Socioeconomic indicators show moderate variation, with ESCS_homeposs (*M* = 1.60, SD = 0.66) and ESCS_Digital_deviceshome (*M* = 1.55, SD = 1.72) indicating some degree of stratification. Moreover, behavioral and school climate variables also vary widely. Truancy (*M* = 18.65, SD = 36.77) and absenteeism (*M* = 9.23, SD = 16.36) suggest uneven student engagement, while school-related measures such as sense of belonging (*M* = 18.59, SD = 3.66) and teacher relations (*M* = 38.22, SD = 2.73) appear more stable.

The data reveal substantial heterogeneity across individual, instructional, and contextual factors, supporting the use of ML approaches to capture complex patterns underlying mathematics performance.

### 4.2 Model performance comparison

Table 3 summarizes the test set performance across all evaluation metrics. XGBoost emerged as the best-performing model (*R*^2^ = 0.5703, RMSE = 65.06, MAE = 51.59), explaining 57.03% of the variance in mathematics performance, representing a substantial improvement over the baseline Random Forest model (*R*^2^ = 0.5152). LightGBM, AdaBoost, Elastic Net, and Linear Regression demonstrated comparable performance (*R*^2^ = 0.5746, 0.5220, 0.1290, 0.1288, respectively), suggesting that gradient boosting methods outperform bagging-based ensemble approaches for this prediction task (see Table 3). The AUC-Regression metric, which captures the cumulative distribution of prediction errors, was highest for XGBoost (0.8399), indicating a greater concentration of errors at lower residual magnitudes. Additionally, all models achieved explained variance scores closely aligned with their respective *R*^2^ values (difference < 0.01).

**Table 3:** Model performance comparison.

Model	*R* ^2^	RMSE	MAE	EV	AUC
Random forest	0.4924	70.72	56.10	0.4926	0.7941
XGBoost	0.5703	65.06	51.59	0.5703	0.8104
LightGBM	0.5700	65.08	51.62	0.5700	0.8050
AdaBoost	0.5220	68.62	54.64	0.5220	0.8360
Elastic net	0.1290	92.62	74.85	0.1292	0.8329
Linear regression	0.1288	92.64	74.83	0.1290	0.8333

**Figure 2:**
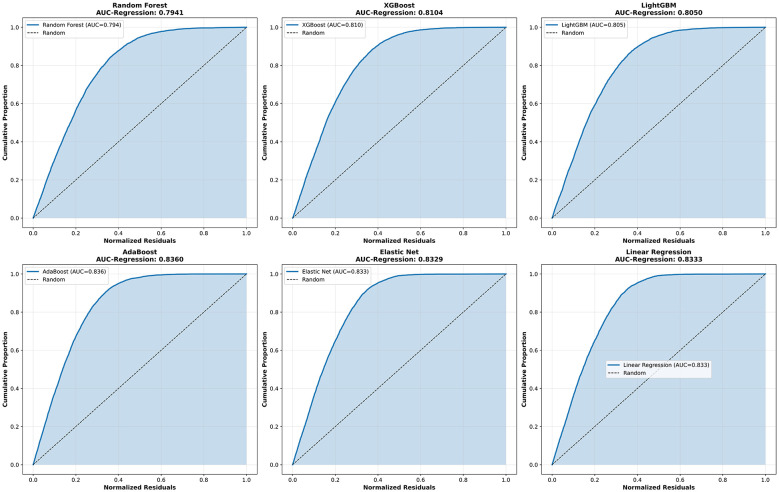
Area under the curve plot.

**Figure 3:**
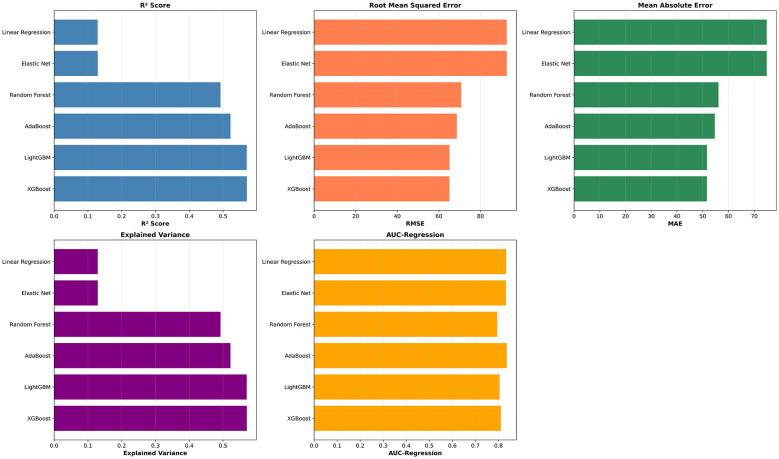
Model performance comparison.

Figure 2 displays AUC-Regression curves for all three models, illustrating XGBoost's superior cumulative error profile compared to the random baseline. Figure 2 also provides a comprehensive visual comparison across all five metrics, highlighting XGBoost's dominance in *R*^2^, explained variance, and AUC, while showing competitive RMSE and MAE performance (see Figures 2, 3).

### 4.3 SHAP analysis results

**Figure 4:**
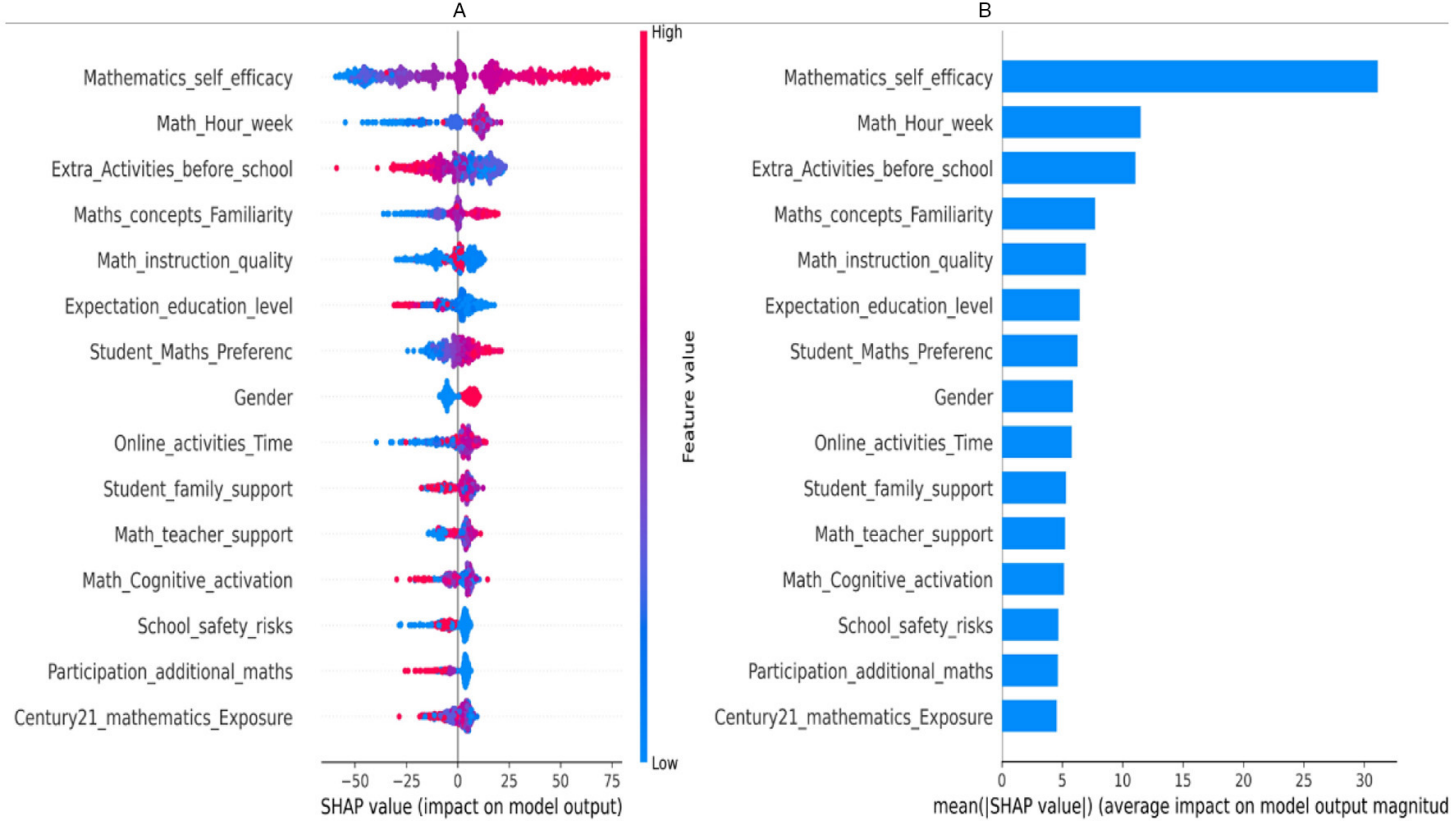
Variable importance and SHAP summary of the model output **(A, B)**.

SHAP (SHapley Additive exPlanations) values were computed to decompose individual-level predictions and quantify both the magnitude and direction of each predictor's contribution to mathematics performance. In the beeswarm plot (Figure 4A), each point represents one student; red points denote high feature values and blue points denote low feature values, while the horizontal position on the *x*-axis reflects whether that feature value pushed the prediction upward (positive SHAP) or downward (negative SHAP). The bar plot (Figure 4B) summarizes the global mean absolute SHAP value per feature, reflecting overall importance irrespective of direction.

Mathematics self-efficacy emerged as the single most influential predictor of mathematics performance, with a mean absolute SHAP value substantially exceeding all other features. High self-efficacy values (red points) were concentrated extensively on the positive SHAP axis, indicating that students with strong mathematical self-belief consistently received upward adjustments in their predicted scores. Conversely, low self-efficacy values (blue points) clustered deeply in the negative SHAP region, demonstrating that poor self-efficacy systematically suppressed predicted performance. The exceptionally wide horizontal spread of this feature's distribution underscores substantial heterogeneity in its impact across individual student profiles. In addition, student mathematics preference exhibited a diffuse distribution near zero with a slight positive tendency. High preference values (red points) were associated with small positive SHAP contributions, while low values (blue points) tended slightly negative, reflecting that intrinsic preference for mathematics exerts a positive but modest independent influence on predicted achievement within this multivariate context.

Educational expectation level exhibited a complex directional pattern. While high expectation values (red points) were generally associated with positive SHAP contributions, notable dispersion across both sides of zero was observed, indicating that the relationship between educational aspirations and predicted performance is not straightforward. This suggests that aspirational motivation alone is insufficient and likely operates through mediating factors such as study behavior, parental support, or institutional context.

ESCS digital devices at home showed a modest positive association, with high values (red points) contributing slightly positively to predicted performance and low values (blue points) associated with small negative adjustments. The narrow SHAP spread indicates that while access to digital devices at home provides a marginal advantage, material access alone appears insufficient to provide meaningful performance gains without accompanying instructional or motivational supports. Furthermore, ESCS home possessions showed a modest yet consistent positive pattern. High values (red points) contributed slightly positively and low values (blue points) were associated with small negative adjustments, indicating that household possessions as a proxy for socioeconomic status exert a limited but directionally positive influence on mathematics performance, likely operating through broader resource availability and learning environment mechanisms.

Mathematics teacher support ranked last among the top 15 predictors in global importance. Its SHAP distribution was narrow and centered near zero, with high values (red points) generating marginally positive contributions and low values (blue points) associated with slight negative adjustments. While directionally consistent with the expectation that teacher support promotes achievement, the modest magnitude of its SHAP values suggests that its effect may be partially mediated by instructional quality and student motivational variables already captured elsewhere in the model. Moreover, mathematics instruction quality showed a positive directional association with performance. High quality values (red points) generated positive SHAP contributions, while low values (blue points) were associated with downward adjustments in predicted scores. This result reinforces the pedagogical evidence linking effective instructional practices to improved student outcomes, even after accounting for individual student-level characteristics.

The SHAP bar plot (Figure 4B) reaffirmed mathematics self-efficacy as the globally dominant predictor by a considerable margin, followed by extracurricular activities before school and hours spent on mathematics per week, corroborating the XGBoost native feature importance rankings and collectively highlighting the primacy of affective, behavioral, and instructional factors in determining mathematics performance across the sampled international student population.

### 4.4 Interaction SHAP values

The following four SHAP interaction scatter plots (panels A1, A2, B1, B2) examine whether gender moderates the relationship between four key predictors, namely math teacher support, student family support, math preference, and math self-efficacy, and predicted mathematics performance. In each panel, the *x*-axis represents the standardized predictor value, the *y*-axis represents the SHAP contribution to the model's prediction, and data points are color-coded by gender (blue = 0, female; red = 1, male). Overlapping color distributions indicate gender-invariant effects, while divergence signals moderation.

#### 4.4.1 Gender × math teacher support → mathematics performance

**Figure 5:**
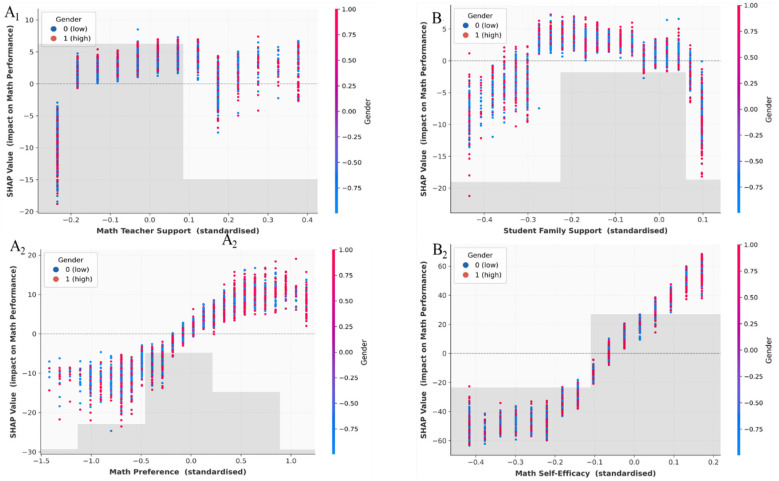
Interaction SHAP values **(A**_**1**_**, A**_**2**_**, B**_**1**_**, B**_**2**_**)**.

Figure 5A1 reveals a non-linear and counterintuitive relationship between math teacher support and mathematics performance. At the lowest support levels (below −0.2), SHAP values descend to approximately −20, indicating that an absence of teacher support constitutes a meaningful penalty on predicted performance. As support approaches the mean, SHAP values recover into positive territory (+1 to +8), reflecting a modest but discernible benefit associated with adequate instructional support. Beyond +0.1, however, returns diminish and values converge toward zero, suggesting that above-average teacher support yields no additional predictive advantage, potentially reflecting compensatory pedagogical responses directed at lower-performing students rather than a genuine performance-enhancing mechanism. Critically, blue and red points are almost entirely superimposed across the full range, providing strong visual evidence that gender does not meaningfully moderate this relationship. Teacher support predicts mathematics performance with comparable magnitude and directionality for both genders, rendering gender a negligible moderator.

#### 4.4.2 Gender × student family support → mathematics performance

Figure 5B1 discloses a complex and non-monotonic pattern between student family support and mathematics performance. At the lowest support values (below −0.4), SHAP contributions are predominantly negative and highly dispersed, reaching as low as −21, signaling that perceived absence of family support strongly suppresses predicted performance. A pronounced upward inflection occurs around −0.2, where numerous points transition into positive SHAP territory (+5 to +7), suggesting a threshold effect whereby even modest improvements from very low support levels yield meaningful predictive gains. Across the middle-to-upper range, SHAP values compress around zero, indicating attenuating and inconsistent effects at average support levels. Regarding gender, blue and red points are broadly co-distributed across most of the range. However, a subtle asymmetry at the lowest support values, where red (male) points concentrate more heavily at extreme negative SHAP scores, tentatively suggests that very low family support carries a marginally greater performance penalty for male students.

#### 4.4.3 Gender × math preference → mathematics performance

Figure 5A2 presents also a strong, consistent, and near-linear positive monotonic relationship between math preference and mathematics performance SHAP values, spanning from approximately −27 at the lowest preference levels to +19 at the highest, a range of 46 SHAP units, establishing math preference as the second most influential predictor in magnitude after self-efficacy. The zero-crossing occurs close to a standardized value of 0, defining a theoretically meaningful threshold: students with average or above-average preference receive a positive model contribution, while those below are penalized. The background histogram confirms that most students cluster between −0.6 and +0.5, yet the SHAP gradient remains steep and consistent across the full distribution. Blue and red points exhibit substantial overlap throughout, confirming broadly gender-invariant effects. Nevertheless, a subtle upward displacement of red (male) points at positive preference values suggests males may derive marginally greater performance benefit from high math preference, a nuance consistent with prior literature on affective engagement and academic outcomes.

#### 4.4.4 Gender × math self-efficacy → mathematics performance

Figure 5B2 unambiguously identifies math self-efficacy as the single most powerful predictor of mathematics performance. The relationship is strikingly linear, steep, and monotonically positive, with SHAP values spanning from approximately −65 to +60—a spread exceeding 125 SHAP units that dwarfs all other predictors considerably. The inflection points fall near a standardized value of −0.1 to 0, where SHAP contributions transition from negative to positive, meaning students at or above average self-efficacy receive a net performance benefit while those below are penalized with escalating severity. This gradient implies that self-efficacy operates as a critical performance-determining variable with broad predictive leverage across the entire student distribution. Most notably, blue and red points are virtually indistinguishable at every point along the continuum, constituting compelling evidence that gender does not moderate the self-efficacy–performance relationship. The transformative predictive power of self-efficacy operates with equivalent force for both male and female students.

Across all four panels, a consistent and theoretically important pattern emerges: gender operates as a negligible moderator of the relationships between the four key predictors and mathematics performance. While subtle gender asymmetries are detectable at the distributional extremes of family support and math preference, these interactions are insufficiently pronounced to constitute meaningful differential effects. The dominant finding is one of gender convergence, the mechanisms through which self-efficacy, preference, teacher support, and family support shape mathematics performance function with broadly equivalent force for both genders. Among the four predictors, math self-efficacy stands as the unequivocal dominant driver, followed by math preference, with teacher support and family support exhibiting more constrained, non-linear, and contextually contingent effects.

## 5 Discussion

### 5.1 Principal findings

This study developed and validated a ML pipeline to predict PISA mathematics performance among East Asian students. After testing six ML models, we have achieved a strong predictive accuracy through an optimized XGBoost algorithm (*R*^2^ = 0.5703, RMSE = 65.06), explaining nearly 57.03% of the variance in mathematics achievement. This performance level is comparable to or exceeds prior ML applications in large-scale educational research, where *R*^2^ values in the range of 0.40–0.55 are commonly reported for student-level outcome prediction using survey-based features. The ability to explain more than half of the variance in mathematics performance using student-level behavioral, affective, and contextual variables is theoretically significant and speaks to the salience of student-proximal factors in shaping academic outcomes. Importantly, these findings are most coherently interpreted through the lens of SDT, which provides a unifying framework for understanding how motivational and psychological factors translate into measurable academic performance.

### 5.2 Mathematics self-efficacy as the dominant predictor

The emergence of mathematics self-efficacy as the most dominant predictor constitutes the theoretically and practically consequential finding of this study. It is worth mentioning that, machine learning-derived feature importance, including SHAP-based rankings, reflects the predictive contribution of a variable within XGBoost model, may different from theoretical primacy, variable is causality, universality. The important is this result is consistent with Bandura's (1997) SCT, Ryan and Deci's (2020) SDT, and previous empirical evidence, which posits that self-efficacy beliefs, individuals' judgments of their capability to execute specific performances, function as proximal determinants of academic motivation, persistence, and ultimately achievement. Within Bandura's framework, self-efficacy does not merely reflect prior performance but actively shapes future behavior through its influence on goal setting, effort regulation, and resilience in the face of difficulty, which in turn contributes to mathematics performance (Van den Bossche et al., 2006).

Moreover, though learning-derived importance could not necessarily equate to theoretical values, mathematics self-efficacy closely aligns with the psychological need for competence, representing students' perceived effectiveness in interacting with academic challenges (Abdulla Alabbasi et al., 2023; Christy and Mythili, 2020; Eccles and Wigfield, 2023). The magnitude of self-efficacy's effect in the present study, vastly exceeding structural, instructional, and socioeconomic predictors, therefore underscores the centrality of competence satisfaction as a foundational predictor of sustained academic engagement and performance (Abdulla Alabbasi et al., 2023). This magnitude warrants particular attention in the East Asian cultural context. In other words, Confucian Heritage Culture (CHC), which underpins the educational philosophies of Chinese Taipei, Japan, Korea, and other sampled economies, traditionally emphasizes effort, diligence, and moral self-cultivation as pathways to academic success (Tweed and Lehman, 2002). Within this cultural framework, students who internalize high self-efficacy beliefs may be particularly well-positioned to harness effort-oriented norms, as their belief in their capability sustains the intensive, self-directed study behaviors that CHC educational environments demand and reward. Conversely, students with low self-efficacy in high-pressure CHC contexts may be especially vulnerable to disengagement or performance suppression, given the acute social consequences of academic failure in these societies, including family honor, university entrance, and long-term occupational prospects (Hau and Ho, 2010).

Furthermore, from an expectancy-value theory perspective (Eccles and Wigfield, 2023), self-efficacy interacts with subjective task value to determine achievement-related choices; in East Asian contexts where mathematics is culturally valorized, high self-efficacy may amplify engagement in ways that compound academic advantages over time, while low self-efficacy may interact with performance-oriented classroom climates to produce particularly pronounced suppression effects.

### 5.3 Time investment variables

Mathematics hours per week and mathematics homework time ranked among the top predictors; however, analysis results revealed important nuances in their directional effects. While higher weekly mathematics time was positively associated with performance, the distribution of SHAP values suggested heterogeneity in this relationship, with some high-engagement students showing only modest contributions. This pattern is consistent with diminishing returns effects and challenges simplistic “more is better” assumptions that pervade East Asian education policy (Jhang and Lee, 2018). In detail, East Asian education systems are internationally recognized for their high instructional time norms. Students in China, South Korea, and Japan routinely spend substantially more hours on academic study, both in formal schooling and private tutoring, than their Western counterparts (OECD, 2018). Yet international evidence increasingly suggests that sheer time investment, in the absence of cognitive engagement quality, does not reliably translate into proportional achievement gains (Scheerens, 2014). The present findings align with this perspective: the positive effect of mathematics time appears to plateau or diversify at higher levels, suggesting that how time is spent, characterized by cognitive activation, deep processing, and formative feedback moderates its effectiveness more than quantity alone.

This non-linearity may also reflect the distinction between controlled and autonomous forms of engagement: time invested under external pressure or obligation may yield weaker returns than time driven by intrinsic motivation and perceived competence. This finding carries direct relevance to the debate on academic workload and student wellbeing in East Asian contexts (Kim and Lee, 2010). Countries such as South Korea and China have implemented policy interventions to limit excessive after-school tutoring hours, reflecting growing recognition that time-intensive study practices can generate psychological costs, including burnout, reduced intrinsic motivation, and heightened anxiety, that ultimately undermine the performance gains they seek to produce (Ra, 2009; Wu et al., 2021).

### 5.4 Extracurricular activities before school

The non-monotonic pattern observed for extracurricular activities before school, with high feature values dispersed across both positive and negative regions, reflects a theoretically complex predictor whose effect cannot be reduced to a simple directional narrative. Within the East Asian educational context, pre-school activities span a wide spectrum, from academically oriented preparatory programs and supplementary tutoring to physical education and arts-based activities. The differential impact of these activity types on mathematics performance is well documented in the educational literature: academically focused pre-school activities may reinforce content knowledge and study habits, whereas activities with no direct academic relevance may displace study time or produce fatigue effects that interfere with classroom engagement (Mahoney et al., 2005).

Activities that are autonomously chosen and intrinsically motivating may foster positive spillover effects on academic engagement, while externally imposed or repetitive academic drilling activities may erode intrinsic motivation over time. Interpreted through SDT, these patterns reflect differences in autonomy satisfaction, including activities aligned with students' interests enhance motivation, whereas controlled participation may undermine it. In East Asian contexts where extracurricular participation is frequently parent-directed and oriented toward academic enrichment rather than personal interest, the motivational quality of such activities may be particularly variable, helping to explain the non-uniform pattern observed.

### 5.5 Mathematics concepts familiarity and instructional quality

The positive monotonic effect of mathematics concepts familiarity on predicted performance aligns with existing literature (Ainjärv and Laas, 2024), which emphasizes the role of prior knowledge as a scaffold for new learning. In mathematics specifically, cumulative knowledge structures mean that deficits in foundational conceptual understanding propagate forward, compounding disadvantage across grade levels. The strong positive contribution of familiarity with core mathematical concepts in the present study reflects this cumulative nature of mathematical knowledge and suggests that early investment in conceptual grounding and procedural drilling alone would yield long-term returns in achievement.

Moreover, mathematics instruction quality similarly demonstrated a positive directional effect, consistent with the extensive pedagogical literature linking teacher effectiveness and instructional coherence to student outcomes (Asiyah et al., 2021; Hattie, 2010). From a SDT perspective, high-quality instruction may function as a need-supportive environment that simultaneously fosters competence through structured learning and relatedness through supportive teacher–student interactions. In East Asian educational contexts, instructional quality is frequently characterized by structured, teacher-directed pedagogy, with strong curriculum alignment and high expectations for student mastery (Leung et al., 2021). While this approach has been associated with high average achievement levels internationally, research also suggests that variation in instructional quality within East Asian systems, particularly between urban and rural schools or between well-resourced and under-resourced institutions, may produce substantial within-system achievement gaps (OECD, 2023). The present finding that instructional quality remains a significant predictor even within a relatively high-achieving regional sample underscores the importance of sustained investment in teacher professional development and pedagogical quality assurance.

### 5.6 Educational expectations and aspirational motivation

Educational expectation level showed a generally positive but heterogeneous directional effect, with notable dispersion values across both sides. This complexity reflects the theoretical tension between aspirational motivation and the psychological costs of high-stakes academic pressure in East Asian contexts. Existing literature suggests that the motivational consequences of high educational expectations depend critically on whether students pursue mastery-oriented goals, focused on learning and self-improvement, or performance-oriented goals driven by external validation and fear of failure (Eccles and Wigfield, 2023). In high-pressure East Asian educational environments, where university entrance examinations function as pivotal life-sorting mechanisms, high educational expectations may be associated with both motivational benefits and anxiety-driven performance suppression if they are not well aligned with students' intrinsic goals. Theoretically, this duality reflects the distinction between internalized aspirations and externally imposed expectations, with the former supporting autonomous motivation and the latter potentially generating controlled regulation and psychological strain.

### 5.7 Online activities and the digital devices use

The predominantly negative directional effect of online activity time at low engagement levels, combined with modest positive contributions at higher levels, reflects what may be characterized as a digital paradox in the East Asian educational context. The baseline negative association between limited online activity and performance may reflect broader patterns of digital resource use, where students with access to and engagement with online educational platforms benefit from supplementary learning opportunities. However, the limited positive effect at high online activity levels suggests that extensive internet use does not straightforwardly translate into academic gains and may reflect the displacement of structured study time by non-academic online activities (Vigdor et al., 2014).

This finding is particularly relevant in the context of the rapid expansion of online educational technology and AI integration in East Asian countries during and following the COVID-19 pandemic, which dramatically accelerated digital learning adoption across the region. While platforms such as online tutoring services, mathematics practice applications, and digital textbooks have proliferated, evidence on their effectiveness remains mixed, with outcome benefits consistently moderated by pedagogical integration quality, student self-regulation, and the socioeconomic context of digital access (Escueta et al., 2020). From SDT understanding, the effectiveness of digital learning environments depends critically on whether they support autonomy, competence, and self-regulated engagement, rather than merely increasing exposure.

### 5.8 Socioeconomic and resource-based predictors

ESCS digital devices at home and ESCS home possessions both demonstrated positive but modest directional effects, consistent with the broader literature on socioeconomic status and academic achievement (Djekourmane et al., 2025; Morkevicius et al., 2022). The limited magnitude of these variables, despite their theoretical relevance, likely reflects the partial attenuation of socioeconomic effects once student-level behavioral and affective variables are controlled, a pattern consistent with research demonstrating that motivational and behavioral factors mediate much of the relationship between SES and academic outcomes (Sirin, 2005). In the East Asian context specifically, where educational investment across socioeconomic strata tends to be high, partly driven by cultural norms around academic achievement as a path to social mobility, the independent predictive contribution of material SES indicators may be more limited than in other regional contexts.

### 5.9 Mathematics teacher support

Mathematics teacher support ranked last among the top 15 predictors, with a narrow distribution centered near zero. While this finding may appear to contradict the extensive literature on teacher-student relationships and their role in academic motivation and achievement (Ansong et al., 2017; Daendliker et al., 2022), it is more appropriately interpreted as evidence of mediation and confounding within the model structure. Teacher support likely affects mathematics performance indirectly by shaping students' self-efficacy, their affective orientation toward mathematics, and the perceived quality of instruction, all of which are included as separate predictors in the model (Li et al., 2021). This implies that, teacher support primarily fulfills the need for relatedness and facilitates the internalization of motivation, rather than exerting a direct effect on performance outcomes. Explicitly modeling these pathways is therefore expected to attenuate the residual direct effect of teacher support on performance.

### 5.10 Understanding SHAP interaction values

Our SHAP interaction findings across all four panels can be meaningfully discussed through SDT (Deci and Ryan, 1985), which posits that human motivation and performance are optimally sustained when three fundamental psychological needs are fulfilled: autonomy (the need to feel volitional agency over one's actions), competence (the need to feel effective and capable), and relatedness (the need to feel meaningfully connected to others). The differential predictive magnitudes observed across the four predictors map coherently onto this tripartite framework.

Specifically, Mathematics self-efficacy, the overwhelmingly dominant predictor, with a SHAP spread around positive side aligns most directly with SDT's competence need. Meaning that, when students perceive themselves as capable mathematics learners, this internal sense of effectiveness fuels intrinsic motivation, deeper cognitive engagement, and ultimately superior performance (Acosta-Gonzaga, 2023). The steep, near-linear SHAP gradient indicates that competence beliefs are not merely facilitative but constitute a foundational psychological prerequisite for sustained academic achievement, consistent with SDT's proposition that competence satisfaction is a central predictor of performance outcomes.

Furthermore, mathematics preference similarly reflects autonomous motivation within the SDT framework. In other words, students who express genuine preference for mathematics are, by definition, operating from intrinsically motivated orientations rather than externally regulated compliance. The near-linear SHAP relationship suggests that this intrinsic affective orientation translates robustly into performance advantages, reinforcing SDT's central claim that autonomous motivation produces more sustained and effective engagement than controlled motivation.

The more modest and non-linear effects of teacher support and family support correspond to SDT's relatedness dimension and broader social-contextual influences. While supportive relational environments are essential for facilitating motivation internalization, SDT posits that such factors operate primarily as enabling conditions rather than direct determinants of performance, which helps explain their comparatively smaller and more context-dependent SHAP contributions.

In addition, interaction patterns suggest that competence and autonomy operate synergistically: high self-efficacy amplifies the positive effects of intrinsic motivation, whereas low self-efficacy constrains them. The consistency of these patterns across gender groups further reinforces SDT's universalist premise that the three basic psychological needs are fundamental across individuals, suggesting that the motivational architecture underlying mathematics performance operates similarly across demographic groups.

### 5.11 Methodological contributions

This study offers several methodological contributions to the application of ML in educational research. First, it implements a comprehensive preprocessing pipeline that integrates median imputation, feature standardization, and post-training feature selection based on importance rankings. This approach provides a principled solution to common challenges in large-scale international survey data, including missing data patterns, distributional heterogeneity, and high dimensionality (Liu et al., 2026). Importantly, training all models on the full set of variables prior to feature selection ensures that importance estimates are derived from the complete multivariate context, rather than being constrained by *a priori* variable reduction.

Second, the use of SHAP values to interrogate model predictions represents a substantive advancement beyond the conventional reporting of aggregate feature importance scores (Shapley, 2016). By enabling the examination of individual-level contributions, directional effects, and distributional heterogeneity, this approach helps bridge the gap between the predictive strength of ML models and the interpretive demands of educational research and policy. Finally, applying this explainable ML framework to East Asian PISA sample contributes to a growing body of literature demonstrating the value of explainable artificial intelligence in education. In contexts where stakeholder accountability and policy relevance are paramount, such interpretable approaches are essential for ensuring that data-driven insights can meaningfully inform decision-making.

### 5.12 Implications for educators and policymakers

While mathematics self-efficacy emerged as a strong predictor in the present analysis, the cross-sectional and non-causal nature of the data necessitates a cautious interpretation of its practical implications. Rather than implying direct causal effects, the findings suggest that self-efficacy is an important correlate of student performance, which may also reflect prior achievement or other unobserved learner characteristics. Accordingly, interventions targeting self-efficacy should be framed as promising but exploratory and should be further evaluated through longitudinal and experimental research designs.

#### 5.12.1 Implications for CHC regional contexts

The findings carry distinctive implications for the East Asian education systems examined in this study, specifically Chinese Taipei, Japan, Korea, Macao, and Singapore, whose educational philosophies are deeply rooted in Confucian Heritage Culture (CHC). These systems share defining characteristics that shape how the present findings should be interpreted: intense examination pressure, high instructional time norms, hierarchical teacher-student relationships, strong family investment in academic achievement, and a cultural emphasis on effort, diligence, and collective academic honor (Tweed and Lehman, 2002).

Within these contexts, the dominance of mathematics self-efficacy as a performance predictor acquires particular significance. CHC educational environments—characterized by high-stakes university entrance examinations and acute social consequences of academic failure, risk cultivating controlled rather than autonomous motivational orientations, where students engage with mathematics primarily out of obligation or fear of failure rather than genuine intrinsic interest (Liem and McInerney, 2018). Professional development initiatives in CHC systems should therefore equip teachers with strategies that actively counteract this motivational climate, including mastery-oriented learning experiences, constructive feedback, peer modeling, and targeted approaches to reducing mathematics anxiety, factors consistently linked to both self-efficacy and achievement (Betoret, 2009; Granziera and Perera, 2019).

School-level interventions such as growth mindset programs (Dweck, 1986) merit careful evaluation within CHC contexts, with attention to cultural adaptation. Their implementation must navigate the tension between CHC's emphasis on effort, which broadly aligns with growth mindset principles, and its simultaneous valorization of performance outcomes. Critically, such interventions should support not only perceived competence but also autonomy and relatedness, consistent with SDT, fostering motivational orientations sustainable under CHC academic pressures.

The non-linear relationship between mathematics time investment and performance carries direct policy relevance across CHC systems, where extended study hours and private tutoring remain institutionally normalized. Existing reform efforts in South Korea and China to regulate excessive tutoring reflect growing recognition that quantity-driven approaches generate psychological costs that ultimately undermine performance (Kim and Lee, 2010). The present findings reinforce this direction: CHC systems should prioritize instructional quality over quantity, emphasizing cognitively engaging, formatively assessed, and autonomy-supportive learning experiences (Boman, 2022; Hou et al., 2023).

Finally, the persistent gender association with predicted performance warrants particular attention in CHC contexts, where gendered expectation structures, including differential parental aspirations and stereotype-consistent teacher feedback, may differentially constrain girls' self-efficacy development. Gender-responsive policies should focus on dismantling stereotype-reinforcing practices and promoting positive mathematical identities among female students, recognizing that observed gender gaps are structurally produced rather than motivationally intrinsic.

#### 5.12.2 Global implications beyond CHC contexts

While the present study focuses specifically on high-performing East Asian systems, its findings carry broader implications for education systems worldwide, though direct policy transfer without local contextual validation should be approached with caution.

The centrality of mathematics self-efficacy as a performance correlate, and its gender-invariant predictive power across the full student distribution, resonates with a robust international evidence base (Bandura, 1997; Pajares and Kranzler, 1995) and suggests that self-efficacy development represents a high-leverage, potentially low-cost intervention target accessible to education systems regardless of resource level. For lower- and middle-income countries where structural investments in educational infrastructure are constrained, the present findings tentatively support prioritizing teacher professional development focused on autonomy-supportive and mastery-oriented pedagogical practices, approaches that do not require substantial material resources but may yield meaningful motivational and performance dividends. However, such implications should be treated as exploratory, pending longitudinal and experimental validation across diverse cultural and socioeconomic contexts.

The finding that instructional quality and mathematics concepts familiarity both demonstrate positive predictive contributions carries universal relevance for systems at varying stages of educational development. The cumulative, scaffold-dependent nature of mathematical knowledge, whereby foundational conceptual gaps compound disadvantage across grade levels, suggests that early investment in conceptual grounding represents a globally generalizable priority, irrespective of cultural context. Education systems experiencing significant within-system achievement gaps, whether driven by socioeconomic inequality, urban-rural disparities, or differential access to qualified teachers, may find particular policy relevance in the present finding that instructional quality remains a significant predictor even within a relatively homogeneous high-achieving regional sample.

The gender-invariant motivational patterns observed across all predictors offer globally relevant guidance for equity-oriented mathematics education reform. Consistent with SDT's universalist premise (Ryan and Deci, 2000; Bureau et al., 2022), the finding that self-efficacy and autonomous motivation operate equivalently for male and female students across CHC contexts suggests that need-supportive learning environments, those that provide all students with genuine competence experiences, autonomous engagement opportunities, and relational support, represent a universally applicable equity strategy. For global education systems seeking to close gender gaps in mathematics participation and achievement, the present evidence implies that intervention efforts should target the equalization of need-satisfying conditions across gender rather than the implementation of gender-specific motivational programs, a reorientation with practical implications for teacher training, curriculum design, and family engagement policy worldwide.

Finally, the integration of psychological, instructional, and contextual variables within a single analytical framework highlights the importance of adopting multidimensional approaches to understanding student achievement globally. Education systems and international development organizations, including those operating within the UNESCO sustainable development goals (SDG 4) agenda, may benefit from moving beyond structural input indicators toward comprehensive assessments that capture student-level motivational and affective factors alongside institutional and socioeconomic variables (UNESCO, 2023). While contextual differences must always be respected and direct policy transfer avoided, the motivational architecture revealed in the present study, with its emphasis on competence, autonomy, and relational support as universal performance determinants, offers a theoretically grounded and empirically informed foundation for educational improvement efforts across diverse global contexts.

### 5.13 Limitations and future research

This study employs a structured analytical pipeline designed to enhance generalizability and replicability across PISA cycles, subject domains, and diverse educational contexts. Nevertheless, several limitations warrant careful consideration and simultaneously point to important directions for future research. First, the cross-sectional nature of the data precludes causal inference. As all variables are measured at a single time point, the analysis captures associations rather than causal relationships. Future research should therefore prioritize longitudinal or panel data, as well as the integration of causal inference methodologies, such as propensity score matching, difference-in-differences, or instrumental variable approaches, to better identify causal pathways and temporal dynamics.

Second, the focus on six high-performing East Asian education systems, many of which are influenced by Confucian cultural traditions, limits the external validity of the findings. These contexts may differ substantially from Western or lower-performing systems in terms of structural conditions, learning behaviors, and policy environments. Future studies should extend the analysis to more diverse international samples to enable systematic cross-cultural comparisons and to assess whether observed patterns, such as the prominence of self-efficacy, are context-specific or more universal.

Third, several key variables, including self-efficacy, parental education, and time spent on mathematics, are based on student self-reports and may be subject to social desirability bias and recall inaccuracies. In addition, important contextual factors, such as teacher quality, peer influences, and neighborhood characteristics, are not captured in PISA microdata, raising the possibility of unmeasured confounding. Future research would benefit from integrating multi-source data, including teacher reports, administrative datasets, and contextual indicators, to improve measurement validity and model completeness.

Fourth, although this study incorporates advanced ML techniques, certain methodological limitations remain. The use of averaged plausible values represents a simplification that does not fully align with OECD-recommended multiple imputation procedures. Future studies should consider more rigorous approaches to handling plausible values. In addition, while ensemble ML models offer strong predictive performance, they are often criticized as “black box” approaches. Although interpretability was enhanced through SHAP and SHAP interaction analyses, further methodological development is warranted, including approaches to better address feature collinearity and to integrate predictive and explanatory modeling frameworks.

Finally, future research should build on the current analytical framework in several ways. Extending the pipeline to additional PISA cycles and subject domains would strengthen replicability. Incorporating school- and teacher-level variables through multilevel ML approaches would enable more comprehensive modeling of nested educational structures. Moreover, further exploration of interaction effects, particularly between theoretically related variables such as self-efficacy, instructional quality, and gender, may provide deeper insights into the conditional mechanisms underlying student achievement.

Despite these limitations, the present study contributes to the literature by integrating ML methodologies with large-scale educational data and offers a multidimensional perspective on the factors associated with student mathematics performance. These findings provide a foundation for more rigorous, theory-informed, and methodologically advanced research in the future.

## 6 Conclusion

This study demonstrates the value of ML, particularly XGBoost for both predicting and interpreting mathematics performance among students in East Asia using PISA 2022 data. By combining strong predictive performance (*R*^2^ = 0.5703) with SHAP-based interpretability, the analysis moves beyond “black-box” modeling to generate theoretically grounded and policy-relevant insights into the determinants of achievement. The findings consistently underscore the predominance of student-proximal factors—most notably mathematics self-efficacy, time investment, and cognitive engagement—over structural and socioeconomic variables. This pattern reinforces the importance of affective and behavioral dimensions of learning within Confucian Heritage Culture educational contexts. Accordingly, interventions likely to yield greater returns than resource-based investments alone include those that strengthen students' mathematical self-beliefs, enhance instructional quality, and promote purposeful and cognitively engaging use of time. At the same time, the study is subject to important limitations, including its cross-sectional design and reliance on self-reported survey measures, which constrain causal inference and may introduce reporting bias. Nevertheless, the results provide a robust empirical foundation for evidence-informed policy and practice in the region. Future research should extend this framework through longitudinal designs, the integration of school- and teacher-level variables, and the application of causal inference methods. Such advances would enable a shift from predictive modeling toward a more profound understanding of underlying mechanisms, ultimately supporting the development of more equitable and effective mathematics education systems globally.

## Data Availability

The data utilized in this paper is openly accessible on the OECD/PISA 2022 database website: https://www.oecd.org/pisa/data/2022database/.
